# Alteration in lipoprotein-associated phospholipase A2 levels during acute coronary syndrome and its relationship to standard biomarkers

**DOI:** 10.1186/1476-511X-11-153

**Published:** 2012-11-10

**Authors:** Petr Ostadal, Dagmar Vondrakova, Andreas Kruger, Marek Janotka, Hana Psotova, Miroslav Prucha

**Affiliations:** 1 Department of Cardiology, Heart Center, Na Homolce Hospital, Roentgenova 2, 150 30, Prague, Czech Republic; 2 Department of Clinical Biochemistry, Hematology, and Immunology, Na Homolce Hospital, Prague, Czech Republic

**Keywords:** Acute coronary syndrome, C-reactive protein, Lipoprotein-associated phospholipase A2, Low-density lipoprotein, Troponin I

## Abstract

**Background:**

Lipoprotein-associated phospholipase A2 (Lp-PLA2) probably plays an important role in the development of acute coronary syndrome (ACS); elevated levels of Lp-PLA2 are associated with a poorer prognosis in patients with ischemic heart disease. Alterations of Lp-PLA2 levels during ACS and its relationship to standard biomarkers are, however, unclear.

**Findings:**

Fifty-one consecutive ACS patients were enrolled in the study. All were managed with early invasive strategy and according to the current guidelines for pharmacotherapy; intensive statin therapy was started in all patients at admission. Serum levels of Lp-PLA2, LDL-cholesterol (LDL), troponin l (Tnl), and C-reactive protein (CRP) were assessed at admission (D0), on the first morning (D1), and on the second morning of hospitalization (D2). Mean serum levels of Lp-PLA2 (ng/mL) decreased from 264.6±19.1 at D0, to 193.2±14.4 at D1 (*P* < 0.001 vs. D0) and 189.8±22.6 at D2 (*P* = 0.002 vs. D0; *P* = not significant vs. D1). Alterations in Lp-PLA2 levels significantly correlated with changes in LDL (r = 0.43; *P* = 0.008). On the other hand, no relationship between Lp-PLA2 and Tnl or CRP was found.

**Conclusions:**

Initially, serum levels of Lp-PLA2 were significantly elevated in ACS patients, but decreased within the first 24 hours after admission and subsequently remained stable. Lp-PLA2 levels correlated with LDL levels but not with Tnl or CRP levels. Our results demonstrated dynamic alterations in Lp-PLA2 levels during the early stages of ACS and, therefore, indirectly support the hypothesis of an active role for Lp-PLA2 in the pathogenesis of ACS.

## Findings

Cardiovascular diseases are the leading cause of death and disability in developed countries; atherosclerosis and thrombosis are the pathogenic mechanisms responsible for the majority of cardiovascular events. Several factors have been hypothesized to participate in the development of atherosclerosis; however, the precise mechanisms of atherosclerotic plaque formation, its vulnerability, and possible disruption remain unclear. Recently, lipoprotein-associated phospholipase A2 (Lp-PLA2) was introduced not only as a risk factor markedly associated with a higher incidence of cardiovascular events, but also as a potentially important pathogenic factor participating in the progression of atherosclerosis [1].

Lp-PLA2 is an enzyme produced by inflammatory cells such as macrophages, foam cells and mast cells, as well as T-lymphocytes in atherosclerotic plaques and by liver cells [2]. Lp-PLA2 is responsible for the hydrolysis of oxidized phospholipids, and leads to the production of highly pro-inflammatory products (lysophosphatidylcholine and oxidized non-esterified fatty acids) [2]. In the blood, Lp-PLA2 is predominantly associated with low-density lipoprotein (LDL) [2]. Lp-PLA2 has been found in both stable and vulnerable atherosclerotic plaques. However, a significantly higher concentration of Lp-PLA2 has been reported in vulnerable and ruptured plaques. In these unstable plaques, Lp-PLA2 is localized not only to the necrotic core, but also in the subendothelial space of regions within the thin fibrous cap or at the site of plaque rupture [3,4].

Numerous epidemiological studies have demonstrated the correlation between elevated Lp-PLA2 levels and increased risk for both primary and secondary cardiovascular events [5,6]. Moreover, although Lp-PLA2 has been shown to be an independent risk factor, enzyme activity and mass positively correlated with LDL-cholesterol and triglyceride levels, and was inversely associated with high-density lipoprotein (HDL)-cholesterol [6]. Several studies have focused on patients with acute coronary syndrome (ACS), usually with a single Lp-PLA2 measurement at enrollment [7-10]. Possible early alterations of Lp-PLA2 levels during the development of ACS and their relationship with standard biomarkers have not, however, been extensively studied.

The study protocol was approved by the institutional ethics review committee, and written informed consent was obtained from all participating subjects. Fifty-one consecutive patients admitted to the Coronary Care Unit for ACS at the Na Homolce Hospital (Prague, Czech Republic) between January and April 2010 were enrolled in the present study. Eligible patients with ST-elevation ACS experienced chest pain at rest less than 12 hours before admission and ≥ 1 mm ST-segment elevation in two or more contiguous leads, or new left bundle branch block on electrocardiogram. Eligible patients with non-ST elevation ACS experienced chest pain at rest during the previous 12 hours and ≥ 1 mm ST segment depression or negative T waves in two or more contiguous leads. All participants were managed with early invasive strategy (ie, they underwent urgent coronary angiography and percutaneous coronary intervention, if necessary). All patients were treated with aspirin and heparin according to the current guidelines for pharmacotherapy, and all patients received clopidogrel immediately. Beta-blockers and angiotensin-converting enzyme inhibitors were administered to patients according to their clinical condition after stabilization; intensive statin therapy was started in all patients at admission. Blood samples for the measurement of Lp-PLA2, LDL, troponin I (Tnl), and C-reactive protein (CRP) levels were drawn at admission (D0), the first morning (D1), and the second morning of hospitalization (D2). Mass measurements of Lp-PLA2 were performed using a commercially available sandwich enzyme immunoassay that is based on two specific monoclonal antibodies (PLAC Test, diaDexus, USA). LDL levels were measured by photometric enzyme assay using the UNICEL DxC 800f Automatic Analyzer System (Beckman Coulter, USA). Tnl was assessed by chemiluminescent immunoassay using the DxI 600f Automatic Analyzer System (Beckman Coulter, USA). CRP was measured using an immunoturbidimetric assay (K-Assay, Kamiya Biomedical Company, USA). Data are expressed as mean ± standard error. Alterations in Lp-PLA2, LDL, CRP, and Tnl levels were analyzed using the Wilcoxon matched-pairs signed-rank test. Correlations of Lp-PLA2 with other biomarkers were evaluated using the Spearman correlation, with *P* < 0.05 considered to be statistically significant.

**Figure 1:**
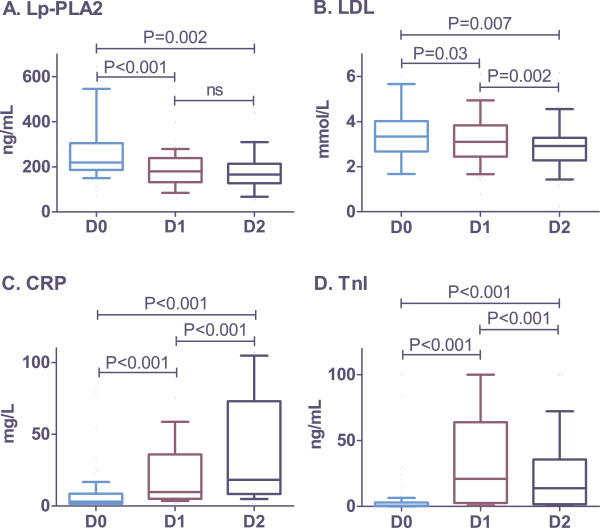
**Alterations in serum levels of Lp-PLA2, LDL, CRP, and Tnl.** Blood samples were taken at admission (D0), first morning of hospitalization (D1), and second morning of hospitalization (D2). The Wilcoxon matched-pairs signed-rank test was used for statistical analysis. Lp-PLA2, Lipoprotein-associated phospholipase A2; LDL, Low-density lipoprotein cholesterol; CRP, C-reactive protein; Tnl, Troponin I.

**Figure 2:**
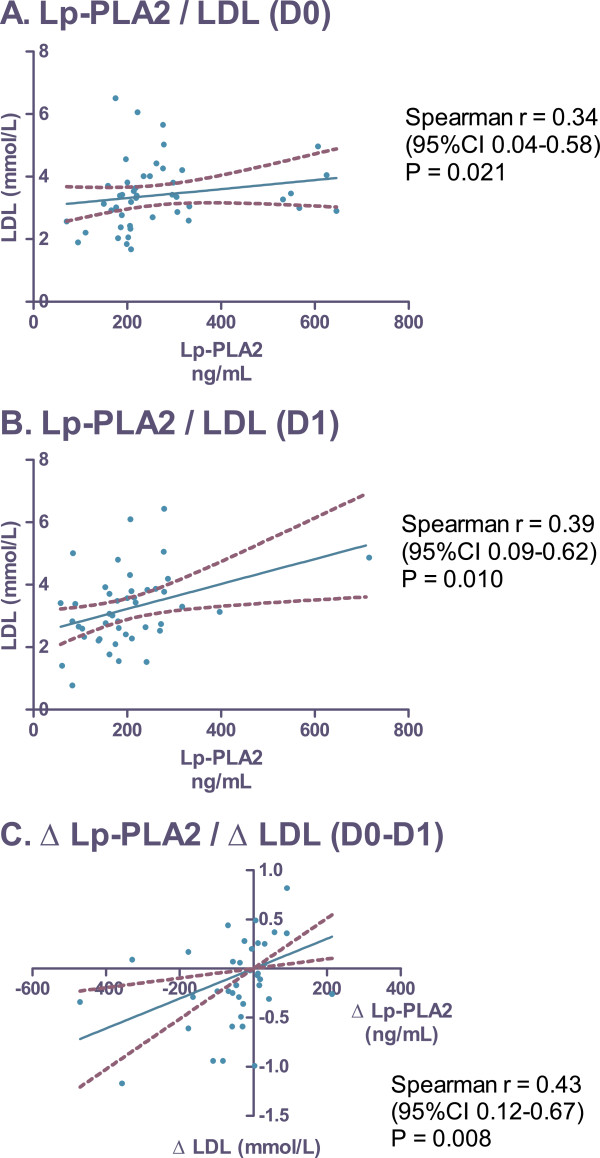
**Correlation of Lp-PLA2 and LDL.** Blood samples were taken at admission (D0), first morning of hospitalization (D1), and second morning of hospitalization (D2). Data were analyzed using the Spearman correlation test. Lp-PLA2, Lipoprotein-associated phospholipase A2; LDL, Low-density lipoprotein cholesterol; Δ (D0-D1), Difference in levels from D0 to D1.

Baseline demographic data, cardiovascular risk factors, and infarction type are shown in Table 1. The mean age of the study population was 62.7 years, the majority of which was male (69%). ST-elevation ACS was experienced by 65% of enrolled subjects (Table 1). The mean serum level of Lp-PLA2 (ng/mL) decreased significantly from 264.6 ± 19.1 at D0, to 193.2 ± 14.4 at D1 (*P* < 0.001 vs. D0) and remain unchanged at D2 (189.8 ± 22.6; *P* = 0.002 vs. D0; *P* = 0.35 vs. D1) (Figure 1A). Alterations in LDL, CRP, and Tnl levels are shown in Figure 1B to D. Significant correlation was found between the levels of Lp-PLA2 and LDL at D0 (r = 0.34; 95% CI 0.04-0.58; *P* = 0.021) and D1 (r = 0.39; 95% CI 0.09-0.62; *P* = 0.01) (Figure 2A to B). Alterations of Lp-PLA2 levels between D0 and D1 positively correlated with the corresponding changes in LDL levels (r = 0.43; 95% CI 0.12-0.67; *P* = 0.008) (Figure 2C). No correlation was found between Lp-PLA2 and CRP or Tnl levels (Table 2).

**Table 1:** Baseline characteristics of the study group (n=51)

	**N**	**%**
Mean age, years	62.7	-
Female	16	31
History of		
CAD	15	29
Diabetes	14	27
Hypertension	34	67
Current smokers	25	49
Type of ACS		
STE	33	65
NSTE	18	35

*CAD*, Coronary artery disease; *ACS*, Acute coronary syndrome; *STE*, ST-elevation on electrocardiogram; *NSTE*, Non-ST-elevation on electrocardiogram.

**Table 2:** Correlation of Lp-PLA2 with other biomarkers

		**Lp-PLA2**			
		**D0**	**D1**	**D2**	**D0-D1**
LDL	r	**0.34**	**0.39**	0.13	**0.43**
	*P*	**0.02**	**0.01**	0.67	**<0.01**
CRP	r	−0.08	0.03	0.01	0.06
	*P*	0.57	0.81	0.82	0.70
Tnl	r	−0.05	0.10	0.08	−0.09
	*P*	0.70	0.49	0.66	0.57

Analysis was performed using Spearman correlation. *CRP*, C-reactive protein; D0 At admission; D1 First morning of hospitalization; D2 Second morning of hospitalization; *LDL*, Low-density lipoprotein cholesterol; *Lp-PLA2*, Lipoprotein-associated phospholipase A2; *Tnl*, Troponin 1.

The major findings of the present study were the rapid changes in Lp-PLA2 levels in the early phases of ACS: elevated levels of Lp-PLA2 decreased during the first 24 hours and subsequently remained stable. Furthermore, we found a significant correlation between Lp-PLA2 and LDL. On the other hand, we were unable to find any relationship between Lp-PLA2 levels and other biomarkers (CRP and Tnl).

To date, several studies have focused on the assessment of Lp-PLA2 in patients with ACS [7-10]; however, none of them used repetitive measurements as in our study. In partial discordance with our results are observations reported by Oldgren et al. [10], who reported that the time delay from symptom onset to blood sampling did not influence Lp-PLA2 levels. We enrolled all participants within 12 hours of symptom onset. Furthermore, the rapid decrease in Lp-PLA2 levels observed in our study were influenced not only by the pathogenic mechanisms participating in the development of ACS, but also by the intensive statin therapy routinely started in our patients at admission. It has been shown that long-term intensive statin therapy may decrease not only LDL levels (by 40 to 60%), but also Lp-PLA2 levels (by more than 20%) [8]. Moreover, it has been reported that the acute effect of statins on LDL is detectable in ACS patients during the first 24 hours after therapy initiation [11,12]. In the present study, both LDL and Lp-PLA2 levels were significantly reduced in the first 24 hours. In contrast to studies of long-term, intensive statin therapy [8], we observed a less marked reduction in LDL levels than in Lp-PLA2 levels.

Our data regarding the correlation of Lp-PLA2 and LDL are in good agreement with other reports. A recently published meta-analysis of clinical trials investigating Lp-PLA2 [6] showed a strong correlation between Lp-PLA2 and LDL, in addition to correlation with other lipid parameters. Similar to our results, the authors of the meta-analysis [6] did not find any correlation between Lp-PLA2 and CRP. On the other hand, a weak, yet statistically significant correlation between Lp-PLA2 and CRP was observed in some other cohorts of ACS patients [10].

The present study has several limitations, most of which were due to the limited size of the study population. It can also be argued that the inclusion of both types of ACS (ST elevation and non-ST elevation) may have increased the heterogeneity of the study group. We included both types of ACS patients based on the same (or at least very similar) pathogenic process in all ACS patients, and on the therapeutic standards in our institution (non-ST elevation patients also undergo urgent invasive therapy).

The results of the present study suggest that dynamic changes in Lp-PLA2 levels during ACS are characterized by a rapid decrease in initially elevated levels. The alterations in the levels of Lp-PLA2 significantly correlated with LDL levels. On the other hand, no association was found between the levels of Lp-PLA2 and CRP or Tnl. Our data provide indirect support for the hypothesis that Lp-PLA2 plays an active role in the pathogenesis of ACS.

## Competing interests

Authors declare that they do not have any competing interests.

## Authors’ contribution

PO: conception and design of the trial, manuscript drafting; DV: laboratory and clinical data analysis and interpretation, manuscript drafting; AK: clinical data analysis and interpretation, critical revision of the manuscript. MJ: laboratory data analysis and interpretation, critical revision of the manuscript. HP: manuscript drafting. MP: conception and design of the trial, critical revision of the manuscript. All authors read and approved the final manuscript.
